# Taxonomic and Metabolic Incongruence in the Ancient Genus *Streptomyces*

**DOI:** 10.3389/fmicb.2019.02170

**Published:** 2019-09-20

**Authors:** Marc G. Chevrette, Camila Carlos-Shanley, Katherine B. Louie, Benjamin P. Bowen, Trent R. Northen, Cameron R. Currie

**Affiliations:** ^1^Department of Plant Pathology, Wisconsin Institute for Discovery, University of Wisconsin-Madison, Madison, WI, United States; ^2^Department of Biology, Texas State University, San Marcos, TX, United States; ^3^Environmental Genomics and Systems Biology, Lawrence Berkeley National Laboratory, Joint Genome Institute, Berkeley, CA, United States; ^4^Department of Bacteriology, University of Wisconsin-Madison, Madison, WI, United States

**Keywords:** *Streptomyces*, metabolism, 16S, phylogenomics, metabolites

## Abstract

The advent of culture independent approaches has greatly facilitated insights into the vast diversity of bacteria and the ecological importance they hold in nature and human health. Recently, metagenomic surveys and other culture-independent methods have begun to describe the distribution and diversity of microbial metabolism across environmental conditions, often using 16S rRNA gene as a marker to group bacteria into taxonomic units. However, the extent to which similarity at the conserved ribosomal 16S gene correlates with different measures of phylogeny, metabolic diversity, and ecologically relevant gene content remains contentious. Here, we examine the relationship between 16S identity, core genome divergence, and metabolic gene content across the ancient and ecologically important genus *Streptomyces*. We assessed and quantified the high variability of average nucleotide identity (ANI) and ortholog presence/absence within *Streptomyces*, even in strains identical by 16S. Furthermore, we identified key differences in shared ecologically important characters, such as antibiotic resistance, carbohydrate metabolism, biosynthetic gene clusters (BGCs), and other metabolic hallmarks, within 16S identities commonly treated as the same operational taxonomic units (OTUs). Differences between common phylogenetic measures and metabolite-gene annotations confirmed this incongruence. Our results highlight the metabolic diversity and variability within OTUs and add to the growing body of work suggesting 16S-based studies of *Streptomyces* fail to resolve important ecological and metabolic characteristics.

## Introduction

From driving the majority of global elemental cycling to mediating the complex interspecies interactions that balance health and disease, the diversity and ecology of microorganisms shapes our world (Falkowski et al., 2008; Lynch and Pedersen, 2016). Advances in molecular and computational techniques have allowed us to uncover the full genetic makeup of organisms across the tree of life and have shed light on their shared and distinct taxonomies, lifestyles, and metabolisms (Louca et al., 2018). Recent studies have found ecology can influence metabolic potential (Chevrette et al., 2019) and gene flow between microorganisms (Smillie et al., 2011). Thus, investigating the interplay between ecology and metabolism is necessary to understand the fitness landscapes influencing microbial evolution.

**Figure 1:**
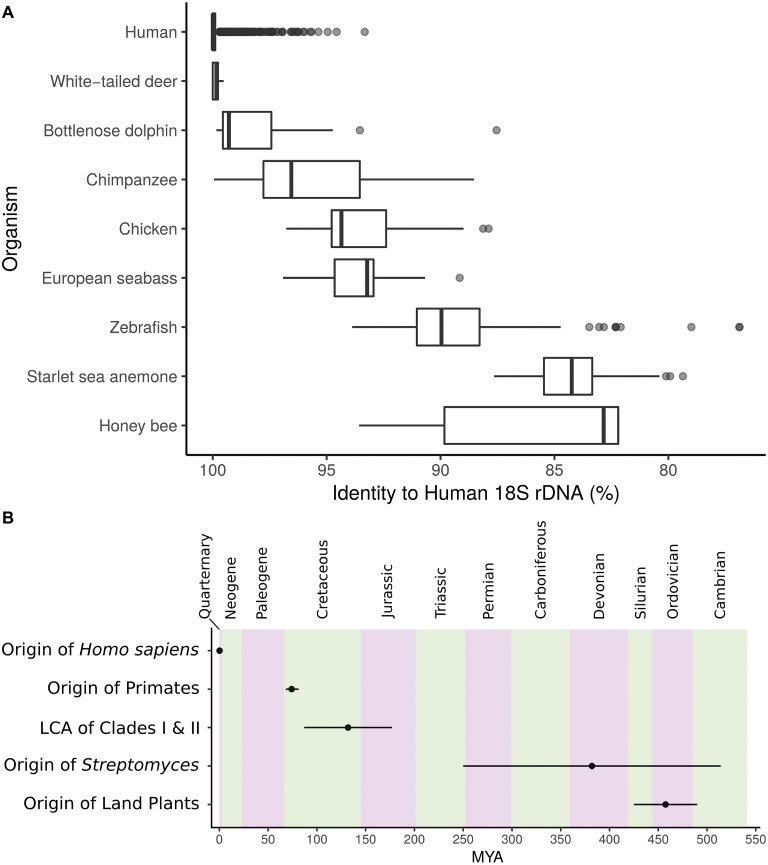
**(A)** Identity to closest human 18S rRNA gene match for several metazoans. Center, median; box, upper and lower quantiles; whiskers, 1.5× interquartile range; points, outliers. **(B)** Timeline depicting the origin of *Streptomyces* and the major split between *Streptomyces* Clades I and II in relation to other events of interest. MYA, million years ago; error bars, lower and upper confidence estimates. Geologic periods are in alternating colors.

In bacterial communities, rather than species as for most plants and animals, the functional units of taxonomy are often defined by operational taxonomic units (OTUs). This is motivated by the absence of morphologically distinguishing traits and the inherent issues associated with bacterial species concepts. Since early studies by Woese and others focused on DNA sequence typing and molecular phylogenetics, the 16S ribosomal RNA (rRNA) gene has been the foundation of microbial ecology and evolution. The vast majority of studies employ this locus as the marker to group bacteria into OTUs, often segregating taxa into groups sharing above 97% 16S rRNA gene sequence identity (Stegen et al., 2013; Ghoul and Mitri, 2016; Kim et al., 2017). However, lateral gene transfer, variable rates of evolution, and dependence upon arbitrary cutoffs greatly impact the 16S gene’s ability to describe shared genomic and metabolic characteristics. Despite high taxonomic diversity as described by OTUs, microbial communities from similar ecologies can exhibit convergence of genomic traits and functional markers (Farjalla et al., 2016). Furthermore, for context, the 18S rRNA gene, the eukaryotic small subunit rRNA with structural and functional homology to 16S in bacteria, is both highly conserved within groups and can show variability that does not necessarily track with evolutionary relationships (Figure 1A). Inconsistent assignment of taxonomic levels confounds the bacterial tree of life; the genetic diversity of the “species” *Escherichia coli* is on the order of many genera. Some genera may encompass hundreds of millions of years of divergence. This has motivated the redefinition of the bacterial tree of life based on genomic similarity at shared loci (Parks et al., 2018). Nevertheless, variable regions have large impacts on metabolism, and thus the fitness, of organisms and metabolic units are often the cargo of lateral transfer (McDonald and Currie, 2017).

As a group, the ecologically and industrially important genus *Streptomyces* is a bacterial taxon commonly found in soil and host-associated microbiomes (Hopwood, 2007; Andam et al., 2016; Chevrette et al., 2019). Originating at approximately the same time period as land plants (Figure 1B; McDonald and Currie, 2017), *Streptomyces* exhibit diverse biosynthetic capacities and their secondary metabolites are the primary source of antibiotics used clinically. Furthermore, *Streptomyces* can produce many other ecologically and medically important bioactive molecules and often dedicate 15% or more of their genomes to secondary metabolism (Chevrette and Currie, 2019). *Streptomyces* genomes are on average 8 megabases (Mb) in length (yet some strains are over 25% larger) and are composed of approximately 72% GC (Hopwood, 2007). While often studied as soil organisms, they readily associate with a diversity of eukaryotic hosts, including many different species of insects (Chevrette et al., 2019). Within *Streptomyces* lineages, diversification of metabolism can coincide with divergence (Choudoir et al., 2018) and variation in metabolic capacity can occur at even identical 16S (Antony-Babu et al., 2017). However, relatively little is known of the phylogenetic and genomic influences on metabolism across the *Streptomyces* phylogeny.

Here, we explore the discordance between taxonomic and metabolic diversity in *Streptomyces* to assess patterns across multiple phylogenetic levels. In 288 finished or near-finished *Streptomyces* genomes, we investigate the relationships between core genome, variable genome, and 16S rRNA gene identity to metabolic and ecologically relevant genetic elements. In a subset of 28 strains distributed across the *Streptomyces* phylogeny, we describe shifts in genomic content in relation to ecology and characterize their exo-metabolomic profiles.

## Materials and Methods

### Data Retrieval

All public *Streptomyces* genomes were retrieved from NCBI on 08/31/2018 and genomes with eight contigs or less were used as part of this study (*n* = 259). 29 additional genomes (28 *Streptomyces* and 1 *Pseudonocardia* outgroup) were sequenced and assembled, herein (see below). Genomes can be accessed at https://bitbucket.org/chevrm/streptomyces_metabolites/.

### Growth Conditions

Mycelial agar plugs (1 mm diameter) were removed from actively-growing YMEA plates of the target strains, and inoculated in 5 ml of Hopwood minimal medium containing 5% (w/v) of glucose (five replicates of each strains). Cultures were incubated for 2 weeks at 30°C in a rotary shaker at 300 rpm. Cultures were centrifuged at 10,000 ×*g* for 10 min, and the supernatants were collected and stored at −20°C.

### Sequencing and Assembly

Cultures were grown in rich medium supplemented with 0.5% glycine and cells were harvested by centrifugation. Cells were washed with 10.3% sucrose, resuspended in lysozyme solution (3 mg ml^−1^ lysozyme, Sigma, in 0.3 M sucrose, 25 mM Tris pH 8, and 25 mM EDTA pH 8), and incubated at 37°C for 30 min. Proteinase K (Thermo Fisher; 20 mg ml^−1^) was added before incubation for 15 min at 42°C. Cells were lysed by adding 2% SDS and rocking for 5 min until lysis was complete. Neutral phenol and chloroform were added, and tubes were gently shaken until uniformly white. After centrifugation, the top layer was transferred to 3 M sodium acetate pH 6 and isopropanol. Tubes were gently mixed until DNA appeared. DNA was pelleted, supernatant was removed, and the pellet was resuspended in TE with 0.2 mg ml^−1^ RNaseA. Tubes were incubated 15 min at 28°C before adding 5 M NaCl and CTAB/NaCl solution. Tubes were incubated for 10 min at 55°C and cooled to 28°C. CHCl3 was added, tubes were gently shaken, and spun for 10 min at 28°C. The top layer was transferred to a new tube and extracted again with phenol and chloroform, followed by extraction with chloroform and precipitation with 3 M sodium acetate pH 6 and isopropanol. The pellet was washed in 70% ethanol and resuspended in water. DNA was quantified, checked for purity, and run on a gel to verify high molecular weight. Genomic DNA libraries for Illumina MiSeq 2 × 300 bp paired-end sequencing were prepared by the University of Wisconsin-Madison Biotechnology Center (TruSeq). Reads were corrected with MUSKET v1.1 (Liu et al., 2013), paired-ends were merged with FLASH v1.2.7 (Magoč and Salzberg, 2011), and assembled with SPAdes v3.11.0 (Bankevich et al., 2012).

### Genomic Characterization

18S rRNA gene sequences annotated as *Homo sapiens* were gathered from the SILVA database (Quast et al., 2012) and aligned against all metazoans in SILVA with blastn (Altschul et al., 1990). Best, non-self hits to human are reported in Figure 1A. Timeline in Figure 1B was created from the following estimates: origin of *H. sapiens* 0.305 million years ago (MYA; 0.26–0.35 MYA low-high estimate) (Schlebusch et al., 2017), origin of primates 74.1 MYA (68.2–81.2 MYA low-high estimate) (Pozzi et al., 2014), origin of land plants 457.5 MYA (425–490 MYA low-high estimate) (Sanderson, 2003), origin of *Streptomyces* 382 MYA (250–514 MYA low-high estimate) (McDonald and Currie, 2017), and lowest common ancestor of the major *Streptomyces* Clades I and II 132 MYA (87–177 MYA low-high estimate) (McDonald and Currie, 2017).

Core genome phylogeny was generated using 93 TIGRFAM proteins in the core bacterial protein set (GenProp0799; http://www.jcvi.org/cgi-bin/genome-properties/GenomePropDefinition.cgi?prop_acc=GenProp0799). Genes were called with prodigal v2.6.0 (Hyatt et al., 2010) and GenProp0799 profile Hidden Markov Models were used to search each genome. HMMER v3.1b2 (Eddy, 2011) was used to identify protein sequences for each protein family. Each family was then aligned using MAFFT v7.245 (Katoh and Standley, 2013). Alignments were then converted to codon alignments and concatenated. The multi-locus phylogeny was generated using RAxML v8.1.24 (Stamatakis, 2014) under the GTRgamma substitution model with 100 rapid bootstraps. Core genome divergence was calculated as genome tree branch lengths scaled from 0 (no distance) to 1 (longest distance).

5S, 16S, and 23S rRNA genes were predicted from genomes with barrnap v0.9 (Seemann, 2014, 2018). Predicted rRNA genes with lengths within 20% of *Streptomyces coelicolor* 5S, 16S, and 23S (117, 1,738, and 3,179 bp, respectively) were aligned with mafft v7.310 (Katoh and Standley, 2013) and nucleotide percent identity at non-gapped alignment columns was calculated between each genome’s rRNA gene. In genomes where more than one 5S, 16S, or 23S rRNA gene was predicted, the highest percent identity match was used.

Genes were predicted with prodigal v2.6.2 (Hyatt et al., 2010) and orthologs were predicted with pyparanoid v0.3.1 (Melnyk et al., 2019). Functional characterization of orthologs was performed by best bitscore of DIAMOND blastp v0.9.24.125 (Buchfink et al., 2015) (query cover of >60%) to the Clusters of Orthologous Groups (COG) database v2014 (Galperin et al., 2015). Resistance elements were predicted with RGI from the CARD database v2.0.3 (McArthur et al., 2013). Carbohydrate active enzyme (CAZy) families (Lombard et al., 2014) were identified with HMMER v3.2.1 (Eddy, 2011) (e-value cutoff 1e−5 for proteins >80aa, 1e−3 otherwise). Metabolic hallmarks were identified from profile hidden Markov models (pHMMs) from Louca et al. (2018) and Anantharaman et al. (2016) following presence/absence requirements in the Louca study. ANI between genomes was calculated *via* fastANI (Jain et al., 2018). BGCs were identified with antiSMASH v4.0.2 (Blin et al., 2017) and grouped into gene cluster families (GCFs) with BiG-SCAPE (Navarro-Muñoz et al., 2018) (distance <0.3).

For a subset of 29 strains, shared gene content was identified and visualized in anvi’o (Eren et al., 2015) *via* the pangenome pipeline (Delmont and Eren, 2018) (minbit = 0.5; mcl-inflation = 4; min-occurrence = 2). Also for these strains, genes were clustered using OrthoMCL implemented in get_homologues (Contreras-Moreira and Vinuesa, 2013). Clustering cutoffs of >75% pairwise alignment coverage and >75% percentage of amino acid sequence identity were imposed. An UPGMA (unweighted pair group method with arithmetic mean) dendrogram was generated from the genome composition presence/absence matrix with Jaccard similarity and 100 bootstrap replications.

### Partial 16S Amplicon Sequencing

Genomic DNA was extracted from isolates using the Powersoil DNA isolation kit according to manufacturer’s specifications (MoBio). Polymerase chain reaction (PCR) was performed using the universal bacterial primer set 27F (5′-AGA GTT TGA TCM TGG CTC AG-3′) and 1,496 R (5′-CGG TTA CCT TGT TAC GAC TT-3′), of hypervariable regions V1-V9. PCR was performed in a standard 25 μl reaction with 1 μl DNA template (20 ng μl^−1^), 12.5 μl EconoTaq (Lucigen corporation), 1 μl combined 10 μM primers, and 12.5 μl water. Initial denaturation for 3 min at 95°C was followed by 3 min annealing at 58°C followed by 35 cycles of 10 s at 96°C and 2 min at 72°C, and a post-cycle extension at 72°C for 7 min. Amplicons were confirmed on 1.2% agarose electrophoresis gel prior to cleanup with Wizard SV Gel and PCR system (Promega). Big Dye sequencing reaction was then performed followed by a secondary clean-up prior to submission to UW Biotech for analysis (University of Wisconsin-Madison). 16S rRNA gene gene fragments were aligned using SINA (Pruesse et al., 2012). A phylogeny was generated with maximum likelihood method and 500 bootstrap replications in the MEGA v6 (Tamura et al., 2013).

### Extraction and LC-MS

Frozen media samples (1 ml) were lyophilized dry, then extracted with 200 μl MeOH containing 10 μM internal standard (5–50 μM of 13C,15 N Cell Free Amino Acid Mixture, #767964, Sigma) followed by vortexing and water bath sonication for 10 min, vortexing again, then sonication for an additional 5 min. After centrifuging at 5,000 rcf for 5 min, supernatant was removed and centrifuge-filtered through a 0.22 PVDF membrane (Millipore, MultiScreen GV Filter Plate, Cat# MSGVS2210) for 3 min at 2,500 rcf into a 96-well plate. Extracts were dried in a SpeedVac and stored at −20°C.

In preparation for mass spectrometry, dried extracts were resuspended in 150 μl MeOH and sonicated in a water bath. Liquid chromatography tandem mass spectrometry (LC-MS/MS) was performed on extracts using an Agilent 1290 LC stack, with MS and MS/MS data collected using a Q Exactive Orbitrap MS (Thermo Scientific, San Jose, CA). Full MS spectra were collected from m/z 70–1,050 at 70,000 FWHM resolution, with MS/MS fragmentation data acquired using 10, 20, and 30 V collision energies at 17,500 FWHM resolution. MS instrument parameters included a sheath gas flow rate of 50 (au), auxiliary gas flow rate of 20 (au), sweep gas flow rate of 2 (au), 3 kV spray voltage, and 400°C capillary temperature. Normal phase chromatography was performed using a HILIC column (Millipore SeQuant ZIC-HILIC, 150 mm × 2.1 mm, 5 μm, Cat# 50454) at 40°C and using a 2 μl injection volume for each sample. The column was equilibrated with 100% buffer B (95:5 ACN:H_2_O w/ 5 mM ammonium acetate) for 1.5 min at 0.45 ml/min, diluting buffer B down to 65% with buffer A (H_2_O w/ 5 mM ammonium acetate) for 13.5 min, down to 0% B over 3 min while increasing flow to 0.6 ml/min, and followed by isocratic elution in 100% buffer A for 5 min. Metabolites were identified based on exact mass and retention time coupled with comparing MS/MS fragmentation spectra to purchased standards.

LC-MS data was analyzed *via* previously described methods (Yao et al., 2015). A set of criteria were used to evaluate each of the detected metabolites and assign a level of confidence in the identification based on (1) absolute value of the difference in retention time from the standard, (2) mass error (ppm) of detected m/z versus theoretical m/z, and (3) MS/MS score comparing experimental MS/MS fragmentation pattern to standard. For retention time and MS/MS comparison, standards were run using the same LC-MS methods as samples. Compounds with the highest level of positive identification had detected m/z <5 ppm from theoretical standard, <0.5 min retention time difference from standard, as well as matching MS/MS fragmentation spectra to either an outside database (METLIN) or internal database generated from standards run and collected on a Q Exactive Orbitrap MS.

For each metabolite present (relative to media controls) in at least three of five replicates, abundances were summed for both positive and negative ion modes and the highest sum was used for subsequent analysis. Genomes and INCHI codes for each strain were then processed with MAGI (Erbilgin et al., 2019). For each metabolite, MAGI scores for each metabolite were then normalized on a scale of 0 to 1 by dividing by the top strain’s MAGI score for a given metabolite. Analysis of variance (ANOVA) of the peak intensity of identified metabolites was performed using MetaboAnalyst 4.0 (Chong et al., 2018). Metabolites with >50% missing values were removed. Remaining missing values were replaced by half of the minimum value in the original data. Peak intensity values were transformed using a generalized log transformation function and scaled using the auto-scaling function. The heatmap was generated using Euclidean distance and Ward clustering algorithm.

Blomberg’s K (Blomberg et al., 2003) with respect to 16S, core genome, and gene composition trees was calculated for each metabolite. Significance (*p*) for each metabolite-tree pair was tested with 1,000 randomizations.

## Results

### Relationship Between rRNA Gene Identity and Core Genome Phylogenetic Divergence

**Figure 2:**
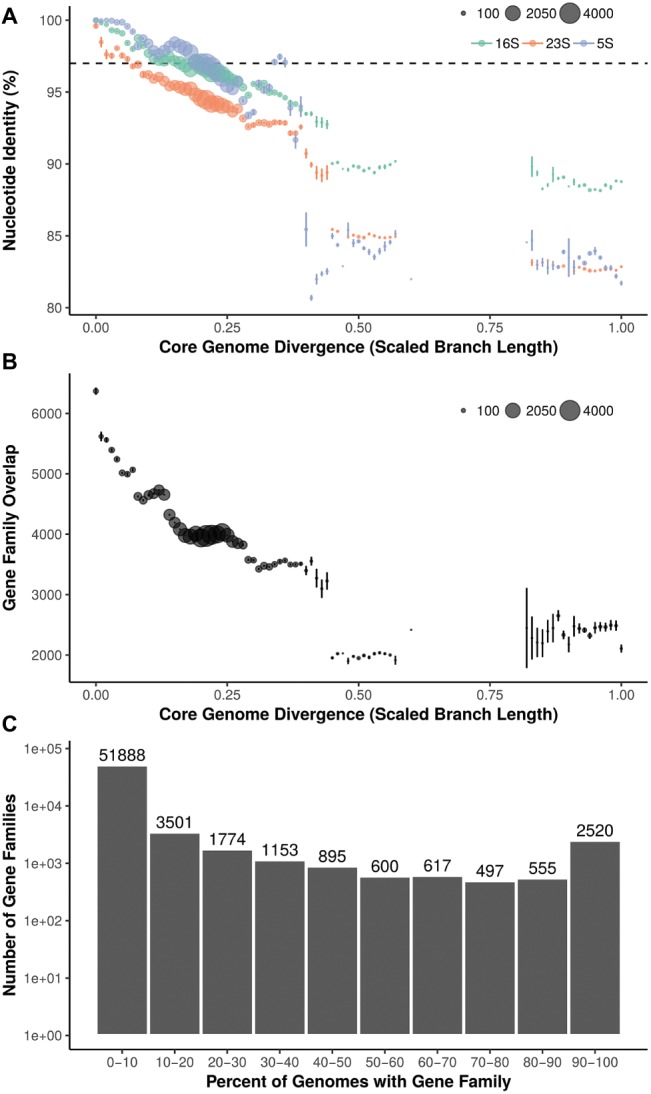
**(A)** Relationship between core genome divergence and 5S, 16S, and 23S rRNA genes in *Streptomyces*. Dashed line = 97%; size of dot = number of pairwise comparisons. **(B)** Relationship between core genome divergence and shared gene families in *Streptomyces*. Size of dot = number of pairwise comparisons. **(C)** Distribution of gene families across the *Streptomyces* phylogeny.

Pairwise 16S, 23S, and 5S rRNA gene percent nucleotide identity was calculated between 288 strains (287 *Streptomyces* and 1 *Pseudonocardia*) and compared to core genome phylogenetic divergence (scaled branch length of phylogeny from 93 conserved bacterial genes) (Figure 2A). 16S rRNA gene similarity ranged from identical (100%) to approximately 88% (*Streptomyces* vs. *Pseudonocardia*). From 0 to ~0.15 core genome divergence, 16S rRNA gene identity drops from 100% to ~97%. This slope is reduced at ~0.15 to ~0.30 divergence where 16S identity remains relatively constant at ~97–96%. *Streptomyces* vs. *Pseudonocardia* comparisons (near 1.0 divergence) are ~88% 16S rRNA gene identity. The longer 23S rRNA gene has a steeper negative slope, with less rRNA gene identity than the shorter 16S or 5S. 5S, the shortest rRNA gene shown, has higher identity at low core genome divergence, but lower identity at higher divergence. Despite a relatively uniform representation of core genome divergence between 0 and ~0.6, all three rRNA genes have a large percentage drop at ~0.4–0.5, 16S from ~93 to ~90%, 5S from ~94 to 85%, and 23S from ~93 to ~90% before another drop to ~85%.

### Ortholog Distribution Relative to Core Genome Phylogenetic Divergence

Within genomes, 64,000 gene families were identified. Overlap in gene families (orthologs) relative to core genome divergence has a greater negative slope than rRNA genes (Figure 2B). Highly similar core genomes (i.e., ~0 core genome divergence) average over 6,000 orthologs shared, but this rapidly decreases until a core genome divergence of ~0.15. Here, the curve assumes an almost step-wise shape with plateaus at ~4,500, ~4,000, and ~3,500 gene families shared at ~0.15, ~0.2, and ~0.3 core genome divergence, respectively. Most gene families (51,888; 81% of orthologs) are found in less than 10% of strains and only 2,520 gene families are shared in 90% or more of the 287 *Streptomyces* (Figure 2C).

### 16S rRNA Gene Identity Versus Average Nucleotide Identity and Ortholog Composition

**Figure 3:**
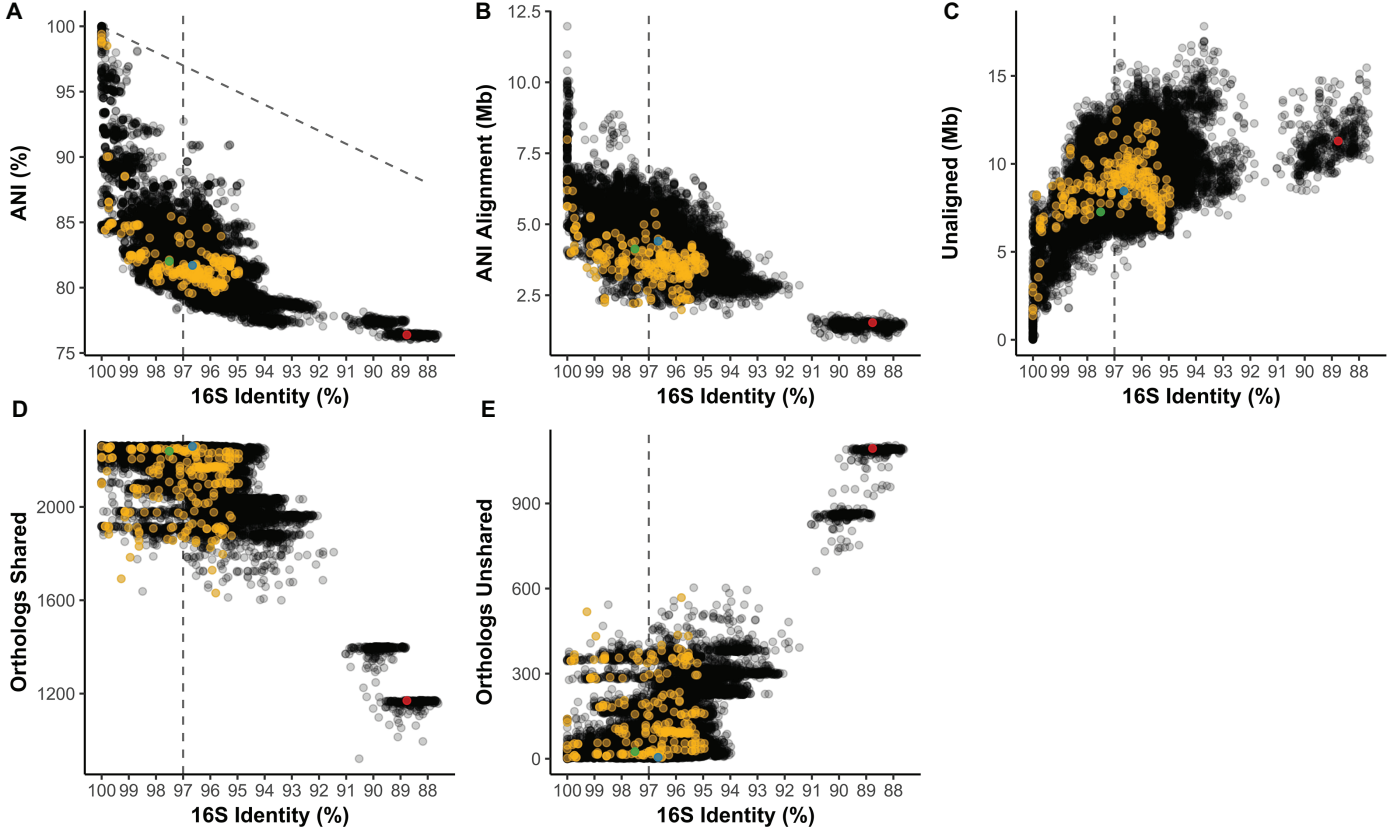
**(A)** Average nucleotide identity (ANI) compared to 16S rRNA gene identity in pairwise comparisons of *Streptomyces*. Diagonal line = 1:1 relationship. Length of the **(B)** ANI alignment length and **(C)** unaligned portions of both genomes compared to 16S identity in the same pairwise comparisons. Number of **(D)** shared and **(E)** unshared orthologs versus 16S identity. **(A–E)** Vertical line = 97% 16S identity; yellow = pairwise comparisons also in the subset from Figures 5, 6. Green = Clade II *S. coelicolor* A3(2) vs. Clade II *S. albus* J1074; blue = Clade II *S. coelicolor* A3(2) vs. Clade I *S. griseus* NBRC 13350; red = Clade II *S. coelicolor* A3(2) vs. *Pseudonocardia* sp. SID8383.

16S identity is not predictive of the average nucleotide identity (ANI) of pairwise aligned regions (Figure 3A). ANI average was 81.69% and ranged from 76.02 to 100%. At 100% 16S nucleotide identity, *Streptomyces* strains can have as low as 84.42% ANI, and across *Streptomyces* ANI and 16S identity do not exhibit a linear relationship (1:1 relationship shown as dotted diagonal, Figure 3A). From identical 16S sequences to the common OTU cutoff of 97% 16S identity (dotted line), ANI can range from 100% to as low as 78.30%, a range only slightly narrower than 100–87.61% 16S identity. *Streptomyces coelicolor* A3(2) (*Sc*) from Clade II of the *Streptomyces* phylogeny had 82.02, 81.70, and 76.38% ANI against *S. albus* J1074 (*Sa*; Clade II), *S. griseus* NBRC 13350 (*Sg*; Clade I), and *Pseudonocardia* sp. SID8383 (outgroup), respectively (Figure 3A). Aligned regions (i.e., shared genetic material) averaged 4.12 Mb and can range from 11.97 Mb to as little as 0.93 Mb (Figure 3B). *Sg* had more aligned genome to *Sc* (4.40 Mb) than did *Sa*-*Sc* (4.13 Mb) despite *Sg* and *Sc* being from different lineages (Clade I and II, respectively; Figure 3B). *Sg*-*Sc* had more unaligned genome (8.45 Mb) than did *Sa*-*Sc* (7.27 Mb) (Figure 3C). At 100% 16S identity, *Streptomyces* can have as much as 8.26 Mb of unaligned, variable content between strains and at the 97% 16S OTU cutoff, this can increase to 13.81 Mb (Figure 3C). Shared gene families ranged from 921 to 2,264 with an average of 2175.5 (Figure 3D). Most *Streptomyces* over 94% 16S identity share over 2,000 gene families (Figure 3D), which is consistent with the “pan” *Streptomyces* genome (i.e., gene families occurring in over 90% of strains) of 2,520 orthologs (Figure 2C). While 16S identity roughly correlates with shared orthologs (Figure 3D), nearly identical strains by 16S (>99% identity) can have as many as 518 unshared gene families (Figure 3E). 16S correlation with presence/absence of gene families belonging to functional categories, including secondary metabolism, shows similar trends (Supplementary Figures S1,S2). *Sg* and *Sc* (Clade I vs. Clade II) have 2,259 shared and only 5 unshared gene families while *Sa*-*Sc* (both Clade II) had 2,239 shared and 25 unshared. *Sc*-*P.* sp. SID8383 had 1,170 shared and 1,094 unshared gene families.

### Metabolic and Functional Genomic Features

**Figure 4:**
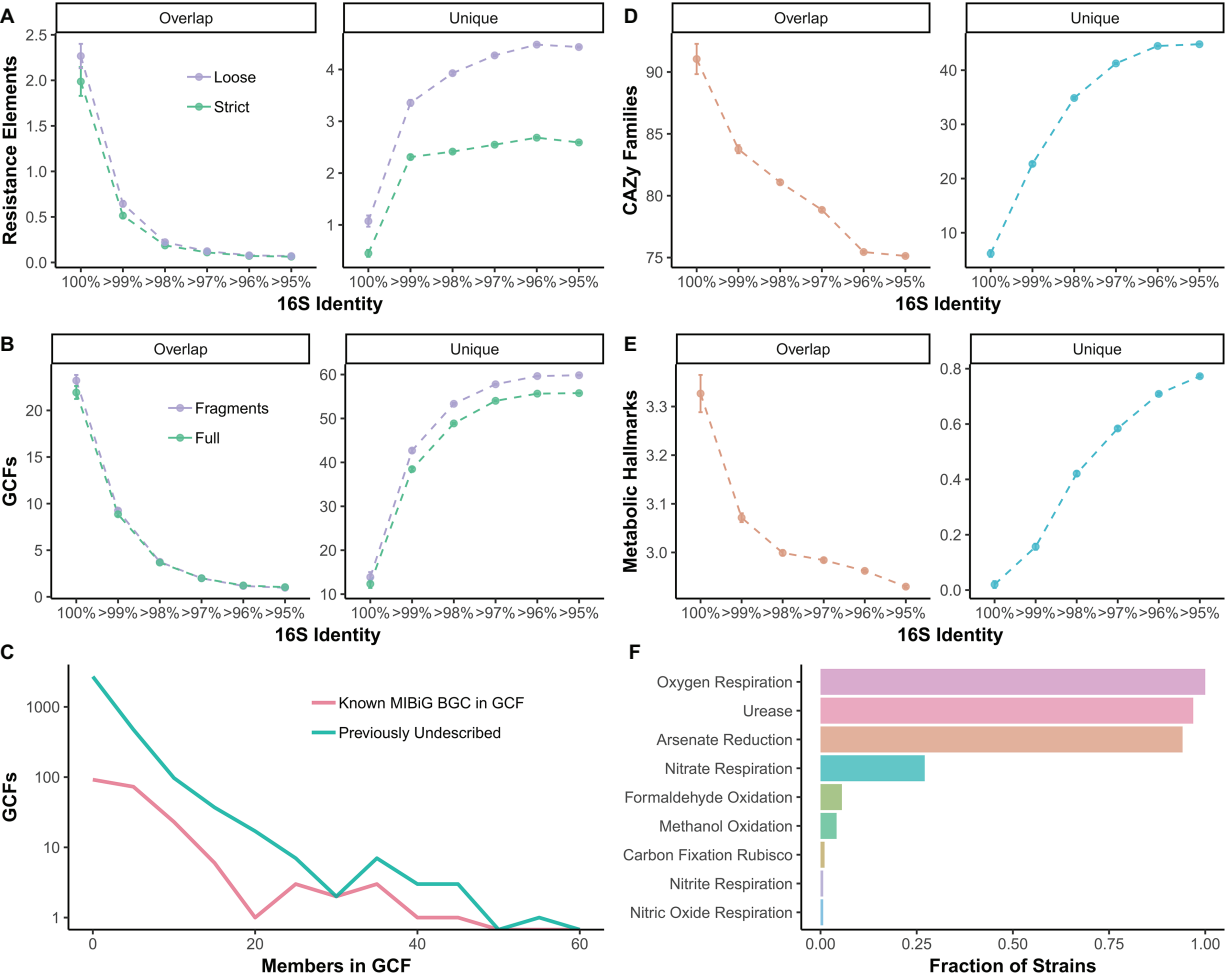
Overlap between strains and unique between strains **(A)** resistance elements, **(B)** biosynthetic gene cluster (BGC) families, **(D)** carbohydrate-active enzyme families, and **(E)** metabolic hallmarks compared to 16S identity thresholds. **(A)** Colors refer to stringency of CARD database matches, either loose or strict. **(B)** Colors refer to either full BGCs (BGCs with 0.5 kb or more flanking contig on both sides) or all BGCs including fragments. **(C)** Frequency of family sizes for gene cluster families. Colors signify whether GCF has a MIBiG match or is previously undescribed. **(F)** Fraction of *Streptomyces* strains with each metabolic hallmark (those with zero not shown).

The amount of shared metabolic and functional genomic features present in two strains is strongly influenced by 16S identity cutoffs (e.g., for an OTU). For example, approximately two elements associated with antibiotic resistance are shared between strains with 100% 16S identity (Figure 4A). However, for *Streptomyces* strains that would group within the same OTU (i.e., >97% 16S identity), average overlap in resistance elements drops to near 0 (Figure 4A). Unique resistance elements are ~2 or ~4 at 97% 16S identity for strict and loose confidence calls, respectively (Figure 4A). Similarly, when biosynthetic gene clusters (BGCs) are grouped into gene cluster families (GCFs), there is a drop from ~21 to ~2 shared GCFs from 100 to >97% 16S identity (Figure 4B). Even at 100% 16S identity, an average of 10 GCFs are unique and increases to over 50 at >97% 16S (Figure 4B). The majority of GCFs have few members, indicating they are rare within *Streptomyces*, but some are seen in over 40 strains (Figure 4C). Most GCFs are previously undescribed, with no BiG-SCAPE (Navarro-Muñoz et al., 2018) matches in the MIBiG database (Figure 4C; Medema et al., 2015). ~92 and ~79 shared carbohydrate-active enzyme (CAZy) families (Lombard et al., 2014) are seen at 100 and >97% 16S, respectively, while ~5 and ~40 unique CAZy families are seen at these 16S identities (Figure 4D). Metabolic hallmarks (e.g., nitrite respiration, arsenate reduction, urease, etc.) range from ~3.3 to ~3.0 shared hallmarks from 100 to >97% 16S (Figure 4E). At 100% 16S, there are on average no differences in metabolic hallmarks between strains, while at >97%, an average of 0.6 are unique (Figure 4E). Most *Streptomyces* exhibit the metabolic hallmarks of oxygen respiration, urease, and arsenate reduction and over a quarter of strains show hallmarks for nitrate respiration (Figure 4F). Formaldehyde oxidation, methanol oxidation, carbon fixation (*via* Rubisco), nitrate respiration, and nitric oxide respiration are rare in *Streptomyces* (Figure 4F).

### Genomic Identity Across Isolate Sources

**Figure 5:**
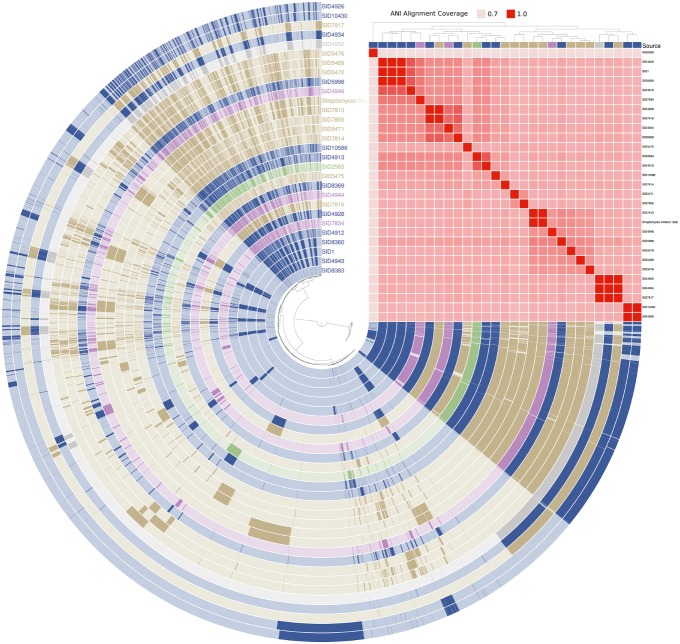
Anvi’o genome map of select *Streptomyces*. Each rail belongs to an individual genome and is colored according to its source (blue = insect-associated; green = plant-associated; purple = fungi-associated; soil = tan; gray = unknown). Presence (dark shading) and absence (light shading) are shown for all gene content in two or more strains. Strains are arranged by ANI alignment coverage and genes are arranged by presence/absence distance (both Euclidean clustering). *Pseudonocardia* SID8383 (inner rail) is used as an outgroup.

The genomic content of a subset of 28 *Streptomyces* strains (shown as yellow in Figure 3) and one *Pseudonocardia* (SID8383) was assessed and visualized in anvi’o (Figure 5; Eren et al., 2015). Strains are marked as either insect-associated (blue), plant-associated (green), fungus-associated (purple), or soil (tan). Dark content in each rail of the genome map represents presence of a gene while light regions signify absence. Genomes are arranged by ANI alignment coverage. Seven of 10 soil strains are found within the same ANI grouping. SID7817 (soil), SID4943 (insect-associated), and SID4952 (unknown source) are closely related by ANI coverage, but exhibit variable genetic regions consistent with Figure 3. The major soil groupings, while clustered together, also show variable content. Only minor differences can be seen between the basal insect-associated grouping of SID4943, SID1, and SID8360.

### Phylogenetic Incongruence to Metabolic Capacity

**Figure 6:**
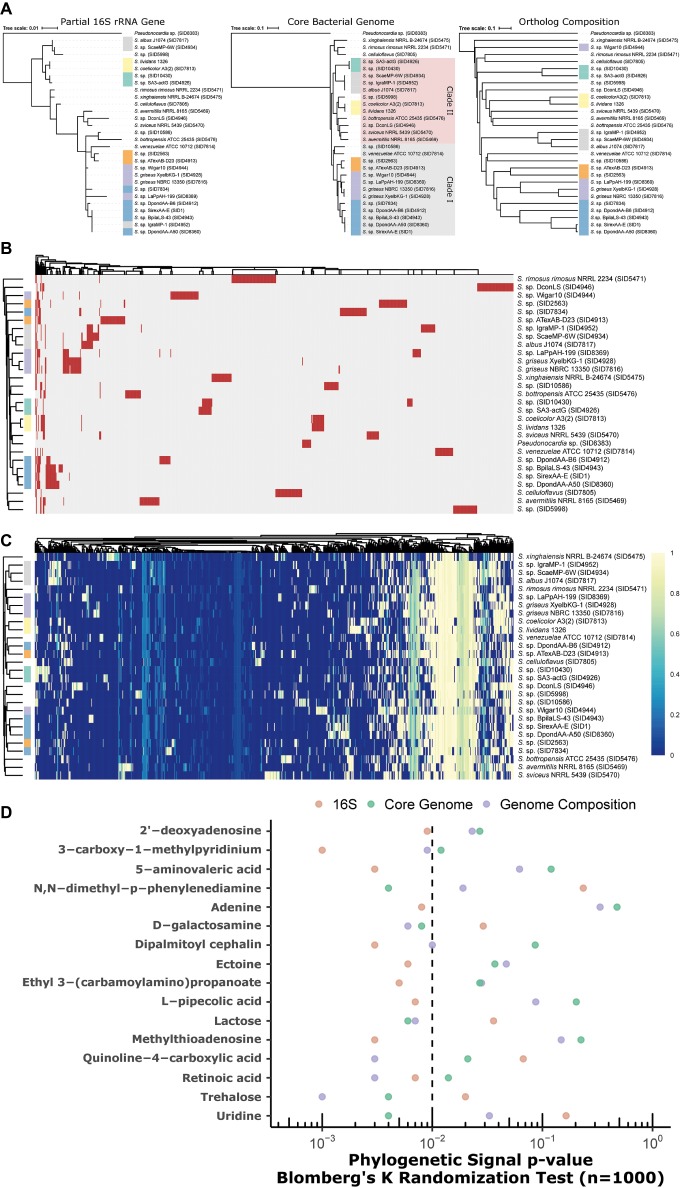
**(A)** Discordant relationships between *Streptomyces* by different methods, partial 16S (V3 and V4), core genome phylogeny, or Jaccard similarity of genome composition (orthologs). **(B)** Presence/absence heatmap of biosynthetic gene cluster families (GCFs) in *Streptomyces*. Rows = strains, columns = GCFs. red = present; gray = absent. **(C)** Genome-metabolite MAGI scores (colors; scaled 0 to 1) in *Streptomyces*. Rows = strains; columns = reactions; clustering = Euclidean. **(B,C)** Annotation bar for strains (colors on row dendrogram) correspond to clades designated in **(A)** above. **(D)** Phylogenetic signal of metabolites with respect to trees in **(A)** above. Significance is marked by *p* < 0.01 (left of the dashed line).

Partial 16S rRNA gene (variable regions V1-V9), core genome, and genome composition (Jaccard similarity) yield discordant phylogenies (Figure 6A). While core genome and composition are the most similar, there is fine-scale discordance in the placement of clades (see green, gray, and SID4944, Figure 6A). Partial 16S gives a low-resolution (and often incorrect) phylogeny, as highlighted by large polytomies and the placement of SID4952 (gray, Figure 6A). Presence/absence clustering of GCFs (Figure 6B) shows some, but not perfect, concordance with core genome groupings. Similar groupings are seen in metabolic pathways through metabolite-gene association MAGI scores (Erbilgin et al., 2019) coupling genomics and metabolomics (Figure 6C, Supplementary Figure S3). A total of 73 metabolites were differentially produced between the strains (ANOVA, FDR < 0.05; Supplementary Figure S3). These metabolites mapped to 63 KEGG pathways in *S. coelicolor* (sco), 59 in *S. avermitilis* (sma), and 57 in *S. griseus* (sgr). Many of these metabolites participate in the metabolism of pyrimidine, purine, “arginine and proline,” “glycine, serine, and threonine”.

Metabolites were tested against 16S, core genome, and genome composition tree topologies for phylogenetic signal. In all cases where a metabolite reached significance (*p* < 0.01) for at least one tree topology, at least one other topology was not significant (Figure 6D). For example, phylogenetic signal for adenine was significant in the 16S tree, but failed to reach significance in both the core genome and genome composition trees. Genome composition was relatively more associated with metabolites for D-galactosamine, quinoline-4-carboxylic acid, retinoic acid, and trehalose. Uridine, lactose, and N,N-dimethyl-p-phenylenediamine best fit the core genome tree. Other metabolites (e.g., L-pipecolic acid, methylthioadenosine, dipalmitoyl cephalin) best fit the 16S tree.

## Discussion

Closely related bacterial species will have genetic and metabolic overlap as dictated by the vertical inheritance of genes (see “overlap” panels in Figures 4A,B,D,E). However, the selective pressures that shape metabolic traits and non-vertical mechanisms of genetic exchange can give rise to unique evolutionary histories. Some traits may be strongly correlated with taxonomy while others may not. Here, we quantify how these shared characteristics correlate with common measures of taxonomic distance within *Streptomyces*. Inferring the lifestyle, metabolism, and ecology of microorganisms from genomic data requires descriptions at finer phylogenetic scales. As a field, microbial ecology is largely built on the foundation of using the 16S rRNA gene as a marker to understand bacterial ecology and evolution (Stegen et al., 2013; Ghoul and Mitri, 2016). Although the limitations of 16S have been recognized (Kim et al., 2017), we believe that better understanding and recognition of these limitations are crucial for the field. Here, we assess these limitations in describing the relationship between metabolism, ecologically important gene sets, core genome, and 16S identity in *Streptomyces*. Through our assessment of genomic and metabolic relatedness across *Streptomyces*, we show that the characters that contribute to metabolic diversity are not fully described by common groupings of taxonomic units. Within *Streptomyces*, 16S identity does not correlate with the most basic ecologically relevant genes, such as CAZy families and antibiotic resistance genes. However, shared content at the tips of the 16S tree (i.e., near identical by 16S) suggests there are groups of *Streptomyces* with highly conserved gene content that relates to ecology. While 16S has some utility in predicting coarse ecological characters (e.g., soil vs. insect-associated in Chevrette et al., 2019) or genus-level taxonomy, phenotypes and patterns that manifest at finer scales may prove elusive, even at high 16S identity. This suggests that if by 16S strains are not identical, an OTU is not a meaningful measure of ecologically relevant diversity. Furthermore, high variability even within identical 16S sequences suggests that future studies should move away from 16S-based descriptions of ecology or metabolic potential.

The advent of 16S sequencing has led to descriptions of microbial environments at unprecedented depth (Moyer et al., 1994; Ferris et al., 1996; Hugenholtz et al., 1998; Gao et al., 2007; Grice et al., 2009), and has even led to the discovery of archaea (Woese and Fox, 1977). These studies have provided the phylogenetic framework from which we define evolutionary relationships across microbial life. However, variation in species- and population-level processes (e.g., specialized metabolism, niche specialization, etc.) is not resolved by 16S identity, as rate of divergence within the 16S does not necessarily correlate with genomic diversity under the influence of unfixed selective pressure. By analogy, at an 18S cutoff of 97% most humans, deer, and dolphins would be grouped into the same OTU (Figure 1A). The diversity within *Streptomyces* presented here suggests we cannot infer lifestyle, function, or ecological phenotype from taxonomic markers.

## Data Availability Statement

The datasets generated for this study and high-resolution versions of each figure can be found in the https://bitbucket.org/chevrm/streptomyces_metabolites/src.

## Author Contributions

MC and CC-S performed genomic analyses and metabolomic analyses. MC, CC-S, and CC wrote manuscript. KL, BB, and TN performed metabolomic experiments.

### Conflict of Interest

The authors declare that the research was conducted in the absence of any commercial or financial relationships that could be construed as a potential conflict of interest.

## Supplementary Material

SUPPLEMENTARY FIGURE S1 Number of shared orthologs at different 16S rRNA gene identities in pairwise comparisons of *Streptomyces*, stratified by COG functional category. Yellow are pairwise comparisons also in the subset from Figures 5, 6; green = Clade II *S. coelicolor* A3(2) vs. Clade II *S. albus* J1074; blue = Clade II *S. coelicolor* A3(2) vs. Clade I *S. griseus* NBRC 13350; red = Clade II *S. coelicolor* A3(2) vs. *Pseudonocardia* sp. SID8383. [**(C)** energy production and conversion; (D) cell cycle control, cell division, and chromosome partitioning; **(E)** amino acid transport and metabolism; **(F)** nucleotide transport and metabolism; **(G)** carbohydrate transport and metabolism; **(H)** coenzyme transport and metabolism; **(I)** lipid transport and metabolism; **(J)** translation, ribosomal structure, and biogenesis; **(K)** transcription; **(L)** replication, recombination, and repair; **(M)** cell wall/membrane/envelope biogenesis; **(N)** cell motility; **(O)** posttranslational modification, protein turnover, and chaperones; **(P)** inorganic ion transport and metabolism; **(Q)** secondary metabolites biosynthesis, transport and catabolism; **(R)** general function prediction only; **(S)** function unknown; **(T)** signal transduction mechanisms; **(U)** intracellular trafficking, secretion, and vesicular transport; **(V)** defense mechanisms; **(W)** extracellular structures].

SUPPLEMENTARY FIGURE S2 Number of unique orthologs at different 16S rRNA gene identities in pairwise comparisons of *Streptomyces*, stratified by COG functional category. Yellow are pairwise comparisons also in the subset from Figures 5, 6; green = Clade II *S. coelicolor* A3(2) vs. Clade II *S. albus* J1074; blue = Clade II *S. coelicolor* A3(2) vs. Clade I *S. griseus* NBRC 13350; red = Clade II *S. coelicolor* A3(2) vs. *Pseudonocardia* sp. SID8383. [**(C)** energy production and conversion; **(D)** cell cycle control, cell division, chromosome partitioning; **(E)** amino acid transport and metabolism; **(F)** nucleotide transport and metabolism; **(G)** carbohydrate transport and metabolism; **(H)** coenzyme transport and metabolism; **(I)** lipid transport and metabolism; **(J)** translation, ribosomal structure, and biogenesis; **(K)** transcription; **(L)** replication, recombination, and repair; **(M)** cell wall/membrane/envelope biogenesis; **(N)** cell motility; **(O)** posttranslational modification, protein turnover, and chaperones; **(P)** Inorganic ion transport and metabolism; **(Q)** secondary metabolites biosynthesis, transport, and catabolism; **(R)** general function prediction only; **(S)** function unknown; **(T)** signal transduction mechanisms; **(U)** intracellular trafficking, secretion, and vesicular transport; **(V)** defense mechanisms; **(W)** extracellular structures].

SUPPLEMENTARY FIGURE S3 Heatmap of average peak intensity for differential metabolites based on ANOVA (FDR < 0.05). The heatmap was generated using Euclidean distance and Ward clustering algorithm.
