# Supplementary figures and images for: Taxonomic and Metabolic Incongruence in the Ancient Genus Streptomyces
Source: Front Microbiol. 2019 Sep 20;10:2170. doi: 10.3389/fmicb.2019.02170 (PMC6763951; doi:10.3389/fmicb.2019.02170)

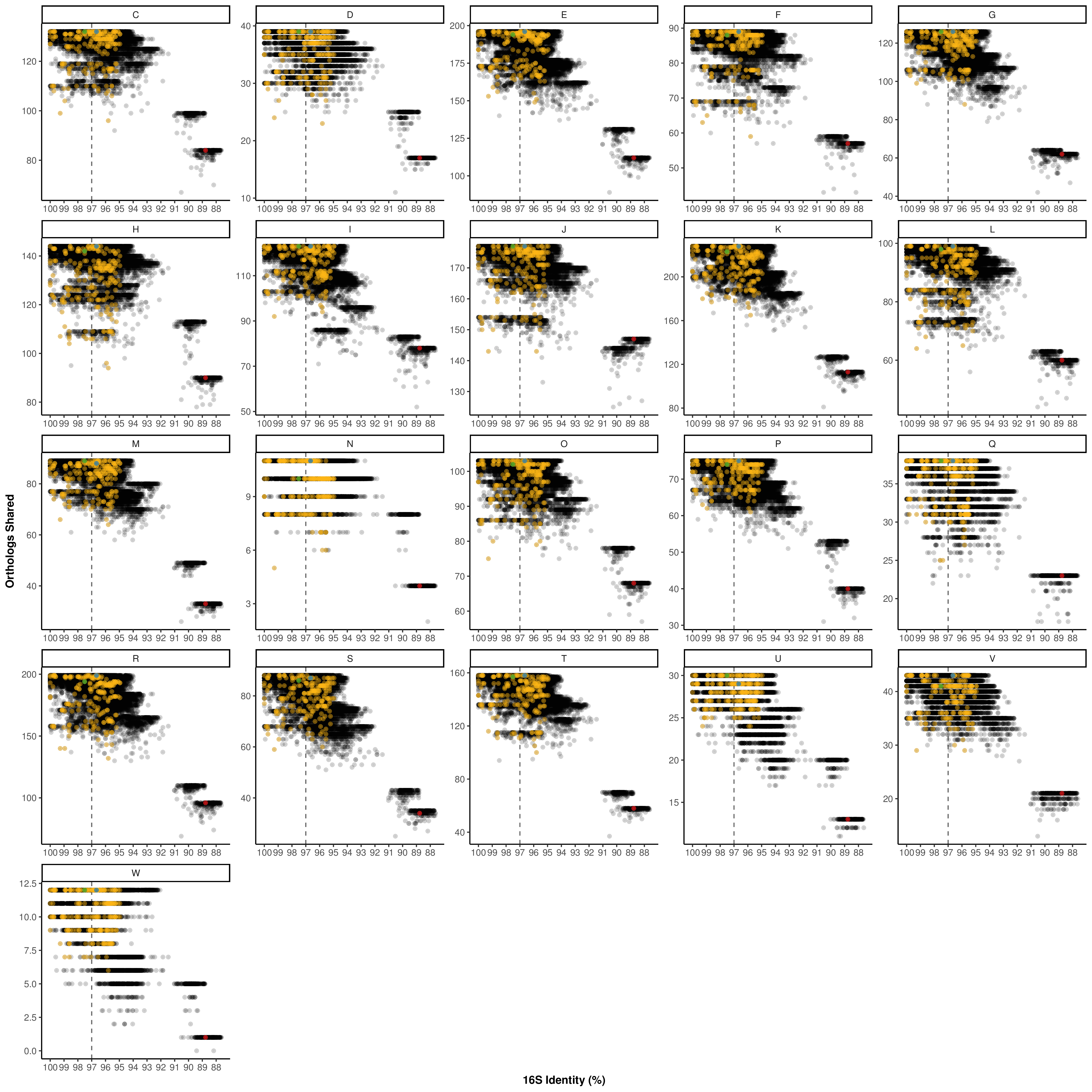

Supplement: SUPPLEMENTARY FIGURE S1 — Number of shared orthologs at different 16S rRNA gene identities in pairwise comparisons of Streptomyces, stratified by COG functional category. Yellow are pairwise comparisons also in the subset from Figures 5, 6; green = Clade II S. coelicolor A3(2) vs. Clade II S. albus J1074; blue = Clade II S. coelicolor A3(2) vs. Clade I S. griseus NBRC 13350; red = Clade II S. coelicolor A3(2) vs. Pseudonocardia sp. SID8383. [(C) energy production and conversion; (D) cell cycle control, cell division, and chromosome partitioning; (E) amino acid transport and metabolism; (F) nucleotide transport and metabolism; (G) carbohydrate transport and metabolism; (H) coenzyme transport and metabolism; (I) lipid transport and metabolism; (J) translation, ribosomal structure, and biogenesis; (K) transcription; (L) replication, recombination, and repair; (M) cell wall/membrane/envelope biogenesis; (N) cell motility; (O) posttranslational modification, protein turnover, and chaperones; (P) inorganic ion transport and metabolism; (Q) secondary metabolites biosynthesis, transport and catabolism; (R) general function prediction only; (S) function unknown; (T) signal transduction mechanisms; (U) intracellular trafficking, secretion, and vesicular transport; (V) defense mechanisms; (W) extracellular structures]. [file Image_1.jpeg]

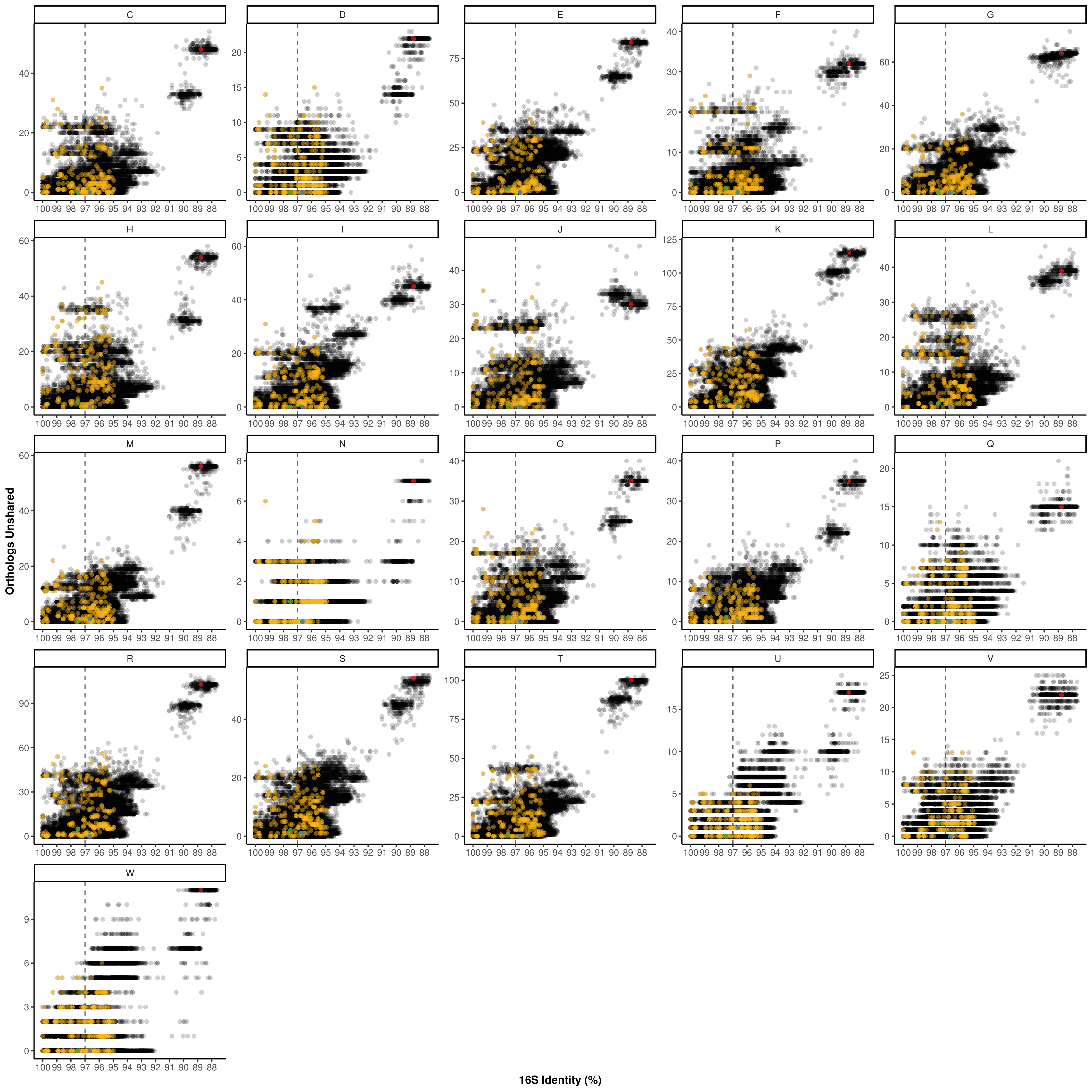

Supplement: SUPPLEMENTARY FIGURE S2 — Number of unique orthologs at different 16S rRNA gene identities in pairwise comparisons of Streptomyces, stratified by COG functional category. Yellow are pairwise comparisons also in the subset from Figures 5, 6; green = Clade II S. coelicolor A3(2) vs. Clade II S. albus J1074; blue = Clade II S. coelicolor A3(2) vs. Clade I S. griseus NBRC 13350; red = Clade II S. coelicolor A3(2) vs. Pseudonocardia sp. SID8383. [(C) energy production and conversion; (D) cell cycle control, cell division, chromosome partitioning; (E) amino acid transport and metabolism; (F) nucleotide transport and metabolism; (G) carbohydrate transport and metabolism; (H) coenzyme transport and metabolism; (I) lipid transport and metabolism; (J) translation, ribosomal structure, and biogenesis; (K) transcription; (L) replication, recombination, and repair; (M) cell wall/membrane/envelope biogenesis; (N) cell motility; (O) posttranslational modification, protein turnover, and chaperones; (P) Inorganic ion transport and metabolism; (Q) secondary metabolites biosynthesis, transport, and catabolism; (R) general function prediction only; (S) function unknown; (T) signal transduction mechanisms; (U) intracellular trafficking, secretion, and vesicular transport; (V) defense mechanisms; (W) extracellular structures]. [file Image_2.jpeg]

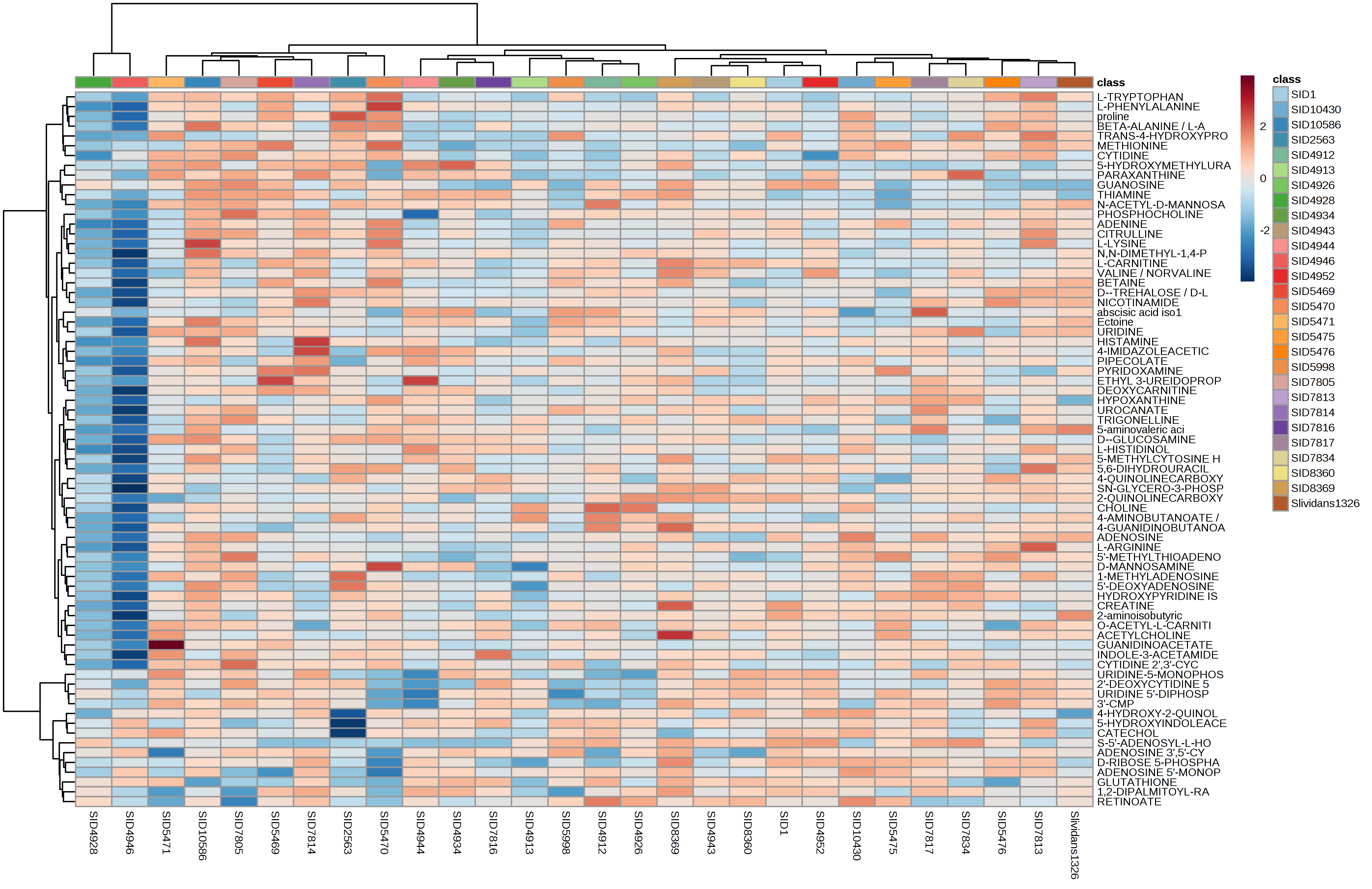

Supplement: SUPPLEMENTARY FIGURE S3 — Heatmap of average peak intensity for differential metabolites based on ANOVA (FDR < 0.05). The heatmap was generated using Euclidean distance and Ward clustering algorithm. [file Image_3.tif]
